# Perceived Risk of Predation Affects Reproductive Life-History Traits in *Gambusia holbrooki*, but Not in *Heterandria formosa*

**DOI:** 10.1371/journal.pone.0088832

**Published:** 2014-02-13

**Authors:** Shomen Mukherjee, Michael R. Heithaus, Joel C. Trexler, Jayanti Ray-Mukherjee, Jeremy Vaudo

**Affiliations:** 1 Department of Biological Sciences, Florida International University, North Miami, Florida, United States of America; 2 School of Life Sciences, University of KwaZulu-Natal, Westville Campus, Durban, South Africa; Ecole Normale Supérieure de Lyon, France

## Abstract

Key to predicting impacts of predation is understanding the mechanisms through which predators impact prey populations. While consumptive effects are well-known, non-consumptive predator effects (risk effects) are increasingly being recognized as important. Studies of risk effects, however, have focused largely on how trade-offs between food and safety affect fitness. Less documented, and appreciated, is the potential for predator presence to directly suppress prey reproduction and affect life-history characteristics. For the first time, we tested the effects of visual predator cues on reproduction of two prey species with different reproductive modes, lecithotrophy (i.e. embryonic development primarily fueled by yolk) and matrotrophy (i.e. energy for embryonic development directly supplied by the mother to the embryo through a vascular connection). Predation risk suppressed reproduction in the lecithotrophic prey (*Gambusia holbrokii*) but not the matrotroph (*Heterandria formosa*). Predator stress caused *G. holbrooki* to reduce clutch size by 43%, and to produce larger and heavier offspring compared to control females. *H. formosa*, however, did not show any such difference. In *G. holbrooki* we also found a significantly high percentage (14%) of stillbirths in predator-exposed treatments compared to controls (2%). To the best of our knowledge, this is the first direct empirical evidence of predation stress affecting stillbirths in prey. Our results suggest that matrotrophy, superfetation (clutch overlap), or both decrease the sensitivity of mothers to environmental fluctuation in resource (food) and stress (predation risk) levels compared to lecithotrophy. These mechanisms should be considered both when modeling consequences of perceived risk of predation on prey-predator population dynamics and when seeking to understand the evolution of reproductive modes.

## Introduction

Predators can affect prey populations through direct consumption (predation) as well as non-consumptive (“risk”) effects [1]–[4]. Risk effects often manifest through changes in foraging behavior and reduced access to energy that decreases reproduction [5]. Less appreciated, but potentially important, is the possibility for physiological stress induced by predator presence to directly impact reproduction [6]–[8].

Reproductive traits of prey individuals can be strongly influenced by predation, either by within-generation responses (plasticity, 9] or intergenerational change in genotypic frequencies presumably because of differential fitness (adaptive evolution, 10]. Examples of plasticity include field studies showing that prey tend to produce smaller clutches in habitats with predators or in the presence of a predation cue [e.g. killifish, *Rivulus hartii*, 11; song sparrow, *Melospiza melodia*, 12, 13]. Other plastic responses to predation risk include shortening of brood retention time [e.g. guppies, *Poecilia reticulata*, 14] and in some extreme cases completely foregoing reproduction [e.g. in Bank voles, *Clethrionomys glareolus*, 15]. Prey may show adaptive genetic differentiation in response to predator presence. In habitats with predators, prey may evolve to allocate more resources per individual offspring [e.g. guppies, *Poecilia reticulata*, 16]. These differences are maintained in common garden experiments, proving they are genetic rather than plastic.

Until now, however, there are relatively few experimental studies on predator-induced breeding suppression [PIBS, e.g. 15, 11, 17, 12, 13, see 18]. Physiological stressors such as predators are among the leading causes of life history variation, with prey responding to stress with higher concentrations of stress hormones (e.g. cortisol level), which in turn affects their immune systems and metabolism, causing suppressed digestion, growth and reproduction [see 3 and 8 for details]. Since PIBS likely affects predator-prey dynamics [19], [3], it is important to test its presence in a broader array of species and contexts.

When exposed to any environmental condition that makes breeding risky in terms of individual survival, behavioral and physiological responses should induce similar responses, namely a change in allocation of ‘effort’ away from reproduction and toward survival, at least in multi-brooded species [20]. Behaviorally, this reallocation is manifested by reduced foraging effort in risky areas or at risky times [see examples in 1], thereby causing a net reduction in energy available to breed and thus to breeding effort. Physiologically, this reallocation makes stressed mothers invest relatively less overall energy in reproduction and relatively more in self-maintenance than would unstressed mothers [20], a standard result in birds and mammals. Thus, the physiological result would mimic energy limitation on reproductive allocation even when females are not proximately energy-limited. All else being equal, and if prey females (fishes in this study) are fed to satiation, we predicted that females exposed to predators should have smaller clutch sizes, and produce larger offspring, compared to those that were not exposed. This would suggest PIBS.

Reptiles and fishes display a diversity of modes of parental care and maternal investment that may affect how stress from predation risk cascades to affecting fitness. In principle, all prey species should be affected by predation risk (as explained above), however there maybe differences in responses due to differences in life history traits related to reproduction. For example, lecithotrophy and matrotrophy are two important reproductive modes. The lecithotrophs differ from matrotrophs is several ways. First, lecithotrophs invest all their reproductive energy in provisioning the eggs ‘up front’, while matrotrophs invest gradually [21]. Secondly lecithotrophs have distinct reproductive episodes that have an ‘all or none’ quality to them, compared to the gradual reproductive output of matrotrophs. Thirdly, lecithotrophs provision their eggs over a much shorter timespan than do matrotrophs. Thus if predation risk were to favor reduced reproductive allocation in both species (see previous paragraph), one would expect to see more distinct tradeoffs between offspring size and number in lecithotrophs, simply because the total investment is limited and ‘paid’ out during a brief time period. Whereas in matrotrophs, investment can accumulate over time (while still being limited) and potential effects of stress (or energy allocation) on embryos can be adjusted through differential gestation length. We tested this hypothesis with two species of fish, *Gambusia holbrooki* (a lecithotroph) and *Heterandria formosa* (a matrotroph). Evaluating how prey species with different reproductive modes (i.e. with different patterns of embryo nourishment, e.g. matrotrophy versus lecithotrophy) may provide insight into how the costs of predation stress are borne by different species.

In this study we provide experimental evidence that predator-induced stress can differently affect reproduction in two species of poeciliid fishes with contrasting reproductive strategies (lecithotrophy and matrotrophy), suggesting that risk effects of predators may directly impact reproductive performance even in the absence of direct energetic costs of anti-predator behaviors, such as time allocation (where, when and how long to forage) and escape behavior. While the effect of predators on prey reproduction has been studied using several different predatory cues [e.g. visual and olfactory –15; auditory –13; chemical –22; simulated predator –23], we tested the effect of only a visual cue.

## Methods

### Ethics Statement

Research was carried out under IACUC Approval # 11-018 of Florida International University.

### Experimental Fishes and their Reproductive Modes

Reproduction in vertebrates can be either oviparous (egg-laying) or viviparous (live-bearing). Viviparous gestation may be either lecithotrophy or matrotrophy, with both reproductive modes found in reptiles and fishes. In lecithotrophic organisms the embryonic development is fueled primarily from yolk, and the mother produces eggs that are stored internally until parturition [24]. In contrast, a matrotrophic species produces ova, and supplements yolk energy after fertilization [25]. Lecithotrophic females allocate energy and materials to developing embryos prior to fertilization, while matrotrophs are able to spread this investment throughout gestation [21]. We used three species of freshwater fishes in our study. *Gambusia holbrooki* (eastern mosquitofish) and *Heterandria formosa* (least killifish), were the two prey fish species, that were visually exposed to their natural predator, the largemouth bass (*Micropterus salmoides). G. holbrooki* is a lecithotroph and lacks superfetation [clutch overlap; 24]. Though post-fertilization provisioning is known in *Gambusia* species [reviewed in 26], its contribution to embryo mass gain during development is small. For example, embryo mass in *G. holbrooki* decreases by as much as 20% or more [e.g., 27], while embryo mass in *H. formosa* increases by as much as 3000% [28], [29]. Thus, *G. holbrooki* and *H. formosa* are representative of the continuum of lethotrophic and matrotrophic poeciliids [26], [30]. Both fishes have relatively short lifespans in the Everglades (approximately a year) [31], [32].

### Experimental Setup

*G. holbrooki* and *H. formosa* were collected from canals bordering the Everglades and ponds on Florida International University’s (FIU) Biscayne Bay Campus. The study did not involve endangered or protected species. Dip nets (5-mm mesh) were used to capture the fishes in the field which were transported to the lab in an insulted container fitted with an aerator. In the lab, fishes (stock) were held in single species groups (75.7 L tanks) for 30 days, before being housed individually for experiments. The fishes were fed *ad libitum* (see species specific food details below) in the morning and in the evening.

**Figure 1:**
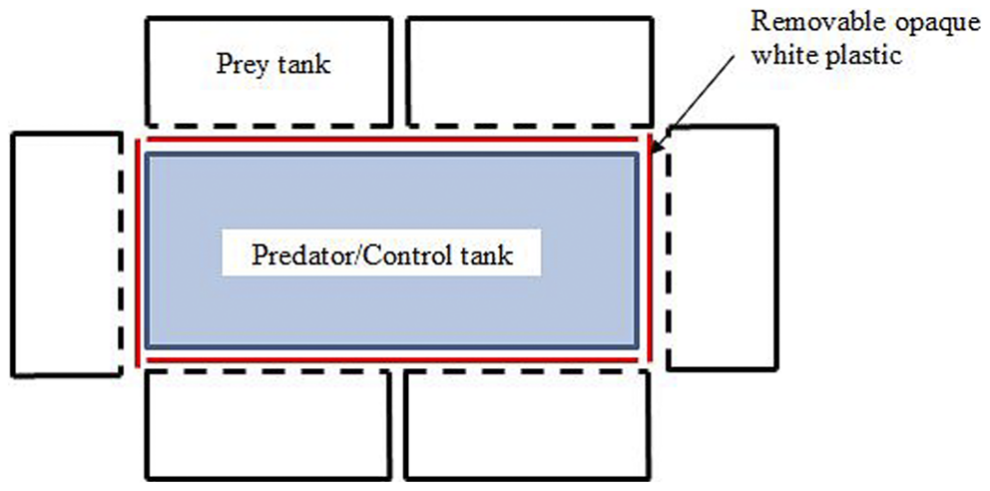
The experimental setup.

The experimental setup (Figure 1) consisted of six replicates of a central 75.7 L tank that contained either a single largemouth bass, *Micropterus salmoides,* (n = 3; standard length, SL: Mean ± SE = 171.1±2.6 mm) with a sponge filter (predator treatment), or a tank with only a sponge filter (control; n = 3). Six 18.9 L tanks (prey tanks, see Fig. 1) were positioned around the central tank, with each containing an adult female *G. holbrooki* (SL: Mean ± SD - Control: 27.55±3.01 mm; Predator: 27.20±2.23 mm) or *H. formosa* (SL: Mean ± SD - Control: 17.01±1.54 mm; Predator: 17.18±1.21 mm).

Each prey tank had an air-stone connected to an aerator, and was covered with white paper on three sides and along the bottom so that the prey fishes could not see movement in the room or other prey fish in adjacent tanks. An opaque white plastic sheet was also placed between the small tanks and the central tank (for both predator and control sets) so that the prey could not see the predator until the initiation of the experiment. All fish were kept on a 12∶12 h light:dark cycle, in a temperature controlled room maintained at 22°C.

Fishes were randomly selected from stock tanks and their weights and standard lengths measured before being housed individually in 18.9 L tanks. From the second day, the opaque partitions were removed twice a day, an hour each in the morning (9–10 AM) and late afternoon (4–5 PM), allowing the prey to see the predator or the control sponge filter. The air supply to the tanks was turned off during these hours. At the end of each hour, the partitions were returned and each prey tank was carefully searched for newborn offspring. As soon as an offspring was found, it was removed, euthanized using an ice slurry [33; approved by FIU’s IACUC], and its SL measured before being individually wrapped in aluminum foil and frozen. Later, offspring were dried for 3 days, and then weighed. Additionally, we also tracked the number of still born neonates present. Reproductive life-history data (litter size, offspring SL, offspring dry-mass) for the first 30 (for *G. holbrooki*) and 28 days (for *H. formosa*) from the start of the experiment were excluded from the analysis since these correspond to gestation time for each species [see 24, 34], and the young born during this time were likely conceived prior to the start of our experimental treatments.

After checking daily for newborns, the fish were fed. Each *G. holbrooki* received 0.05 ml of specially prepared liver paste [35], while each *H. formosa* received a chip (avg. weight - 4.1 mg) of TetraMin tropical crisps (Tetra Holding U.S. Inc.). Bass were fed with one pellet of Tetra Cichlid Jumbo Stick (Tetra Holding U.S. Inc.) at the end of each day. All prey fishes, regardless of treatment, were fed to satiation (indicated by remaining food) in the absence of predator cues. This allowed us to remove any potential effects of predators on energy intake, which might result in differences in reproduction. Experiments with *G. holbrooki* ran between May-August 2011, a time period that allowed us to collect two litters from most individuals, while experiments with *H. formosa* were conducted between September and October 2011.

Standard length of maternal fish was significantly correlated with litter size, but not with offspring SL or offspring dry weight in *G. holbrookii.* Hence we used a linear mixed model (random effects, with a repeated statement) in PROC MIXED [36], to analyze differences in litter size (length as a covariate), offspring SL and offspring dry weight, between treatment (control vs predator) and litter (first vs second litter). For *H. formosa*, we did not find a significant correlation between maternal SL and litter size, offspring SL or offspring dry weight. Since we had only two weeks of data for *H. formosa,* a one-way ANOVA was conducted to test for differences in the number of offspring, offspring SL and dry mass between treatment.

Differences in growth of the experimental (maternal) fishes were evaluated by measuring the SL of the fishes at the beginning and end of the experiment. With initial SL as a covariate, a one-way ANOVA was conducted to analyze differences in growth between treatments for *G. holbrooki*. For *H. formosa*, since there was no significant correlation between SL and growth, a one-way ANOVA was carried out. We used a t-test to calculate inter-brood interval (the number of days between 1^st^ and 2^nd^ litter) difference for *G. holbrooki*. All statistical analyses were conducted in SAS [36].

## Results

### Litter Size

**Figure 2:**
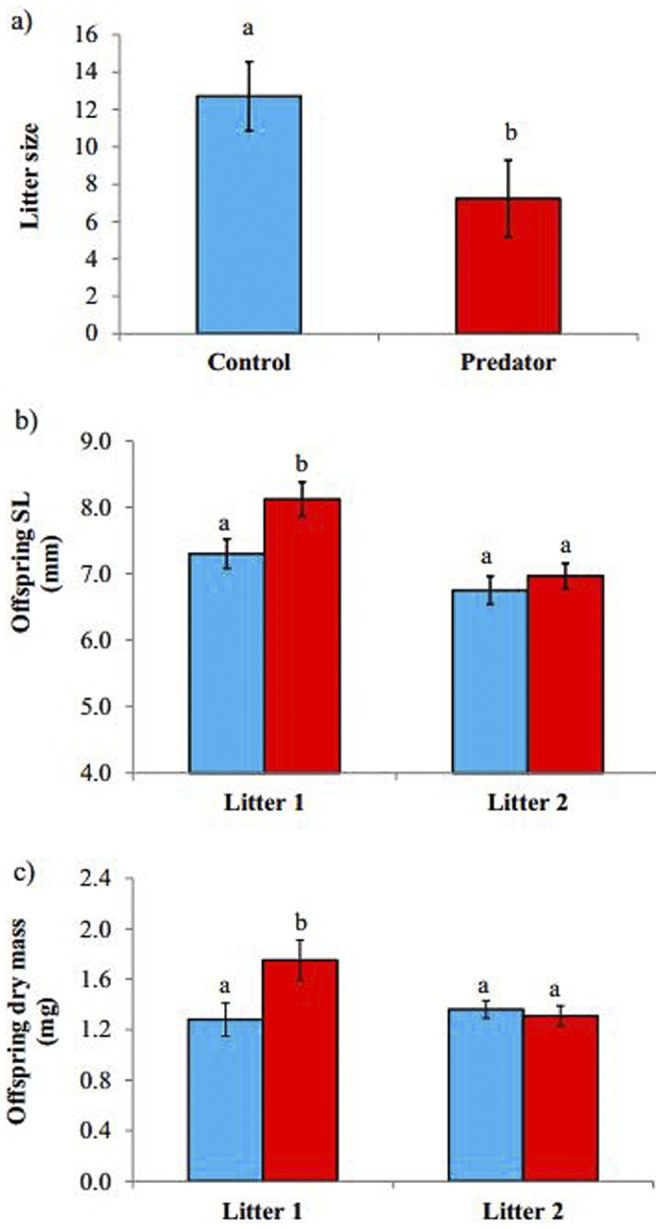
Effect of perceived predation risk on (a) litter size, (b) standard length, and (c) dry mass, in *Gambusia holbrooki*. Red bar indicated predator treatment and blue bar indicate control. Alphabets in figures (b) and (c) indicate within litter differences.

Exposure to predator visual cues modified reproductive parameters in *G. holbrooki* but not in *H. formosa*. *G. holbrooki* exposed to predators produced 43% fewer offspring compared to control individuals (Treatment: P = 0.02, Table 1; Effect size – Cohen’s d = 0.64, effect-size *r* = 0.31, Fig. 2a). Overall litter size of *G. holbrooki* remained the same between first and second litters (Litter: P = 0.64, Table 1). Interestingly, there was no effect of predator treatment in *H. formosa*. Control and predator exposed fishes produced similar number of offspring in both weeks of the experiment (P = 0.28, Table 1).

**Table 1:** Factors affecting reproductive life-history parameters of *Gambusia holbrooki* and *Heterandria fermosa*.

Species	Dependentvariable	Effect	*F*	*P*
*G. holbrooki*	Litter size	Treatment(control, predator)	*F* **_(1,12) = _**7.85	0.02
		Litter(1^st^, 2^nd^)	*F* **_(1,12) = _**0.23	0.64
		Treatment*Litter	*F* **_(1,12) = _**0.01	0.94
	Offspring standardlength	Treatment(control, predator)	*F* **_(1,13) = _**8.56	0.01
		Litter(1^st^, 2^nd^)	*F* **_(1,13) = _**38.72	<0.001
		Treatment*Litter	*F* **_(1,13) = _**3.67	0.08
	Offspringdry mass	Treatment(control, predator)	*F* **_(1,9) = _**3.66	0.09
		Litter(1^st^, 2^nd^)	*F* **_(1,9) = _**2.28	0.17
		Treatment*Litter	*F* **_(1,9) = _**4.73	0.06
*H. formosa*	Number ofoffspring	Treatment(control, predator)	*F* **_(1,21) = _**1.25	0.28
	Offspring standardlength	Treatment(control, predator)	*F* **_(1,22) = _**0.03	0.87
	Offspringdry mass	Treatment(control, predator)	*F* **_(1,22) = _**1.24	0.28

### Offspring Characteristics

Predator-exposed *G. holbrokii* produced offspring that were longer (Treatment; P = 0.01, Table 1; Effect size – Cohen’s d = 0.56, effect-size *r* = 0.27, Fig. 2b) and heavier (particularly in the first litter, Treatment*Litter; P = 0.06, Fig. 2c, Table 1). In the matrotrophic *H. formosa*, neither offspring standard length, nor dry mass was affected by predator treatments (Table 1).

### Other Life-history Parameters

There was no statistical difference in growth between control and predator-exposed individuals in either *G. holbrooki* (ANCOVA, F_1,32_ = 0.05, P = 0.82) or *H. formosa* (ANOVA: F_1,34_ = 3.38, P = 0.08) during the experiment. Predation risk did not affect interbrood interval in *G. holbrooki* (t-test: *t* = 0.15, *P* = 0.89).

### Stillbirth

**Figure 3:**
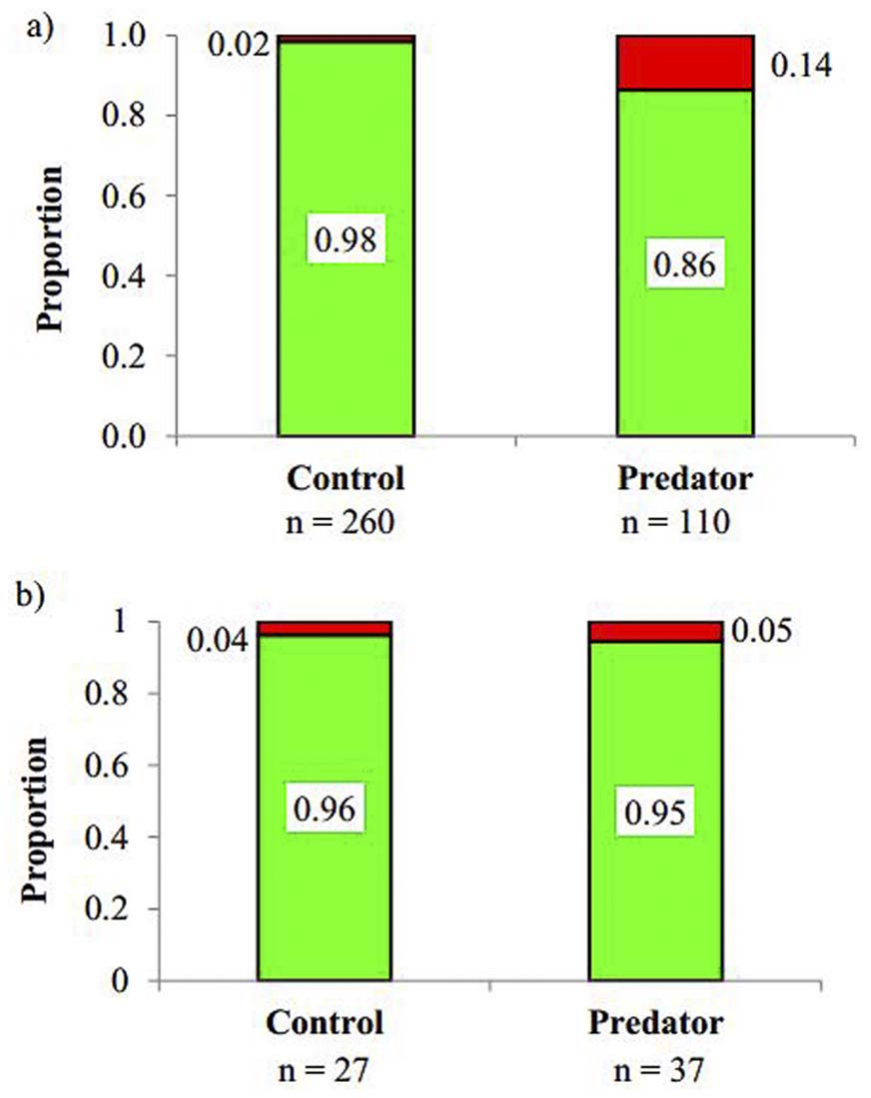
Difference in the ratio of proportion stillbirth (red) to livebirth (green) in the control and predator-exposed treatment. (a) A significant difference in response in the lecithotrohic *Gambusia holbrooki*, and (b) No signifcant difference between treatments in the matrotrophic *Heterandria formosa*. Numbers within, or next to the bars show proportions.

Our treatment affected the ratio of live vs stillbirth offspring for the lecithotrophic prey but not the matrotroph. While *G. holbrooki* mothers that were exposed to predators produced higher proportion (0.14) of stillborn compared to control mothers (0.02; 2×2 contingency Chi-square test, χ^2^ = 23.22, P<0.001; Fig. 3), no such difference was found in *H. formosa* (P = 0.75).

## Discussion

Predation risk may affect prey reproduction through two mechanisms. The first involves reductions because of foraging constraints when threatened by predators. In general, prey reduce their foraging rates, or alter habitat use, in order to enhance safety at the expense of energy intake. Such behavioral changes can result in less energy available for reproduction [2], [37]. Alternatively, in species with parental care, individuals that must forage more cautiously when predators are present may reduce their visits to offspring, reducing offspring survival probabilities [13]. Stress effects of predators may also be important to prey reproduction, even if prey are able to obtain similar amounts of energy compared to risk-free conditions because prey may alter their physiology to promote survival rather than reproduction [3], [38], [8], [39]. We cannot exclude the possibility that our results resulted from differences in feeding, where the predator exposed fishes may have feed less, because we did not measure the amount of unconsumed food each day. However, since we did not find a difference in growth between predator and control fishes, and because predator treatment fishes had several predator (and competitor) free hours to feed, it is most likely that our observed reproductive impacts of predators arose through physiological (stress) responses. Future work should measure both unconsumed food and indicators of stress, such as cortisol.

Predation stress can affect reproduction in prey. Over two decades ago, Ylönen’s study [15] on bank voles (*Clethrionomys glareolus*) provided the first empirical evidence of breeding suppression by a predator through stress. Field data also suggest that stress effects of predation risk could be an important factor in population dynamics. For example, snow shoe hares (*Lepus americanus*) have high cortisol levels [3], [7] and low testosterone responses in the presence of predators [3], while elk (*Cervus elaphus*) have lower progesterone levels that appear to reduce reproductive output in the presence of risk from wolves [4]. Physiological effects of risk could also be maternally transmitted to offspring [40], [41], [23], which could further affect population dynamics.

Typically, seasonal breeders or species with short lifespan resist acute stress while favoring reproduction [5]. Predator exposed *G. holbrooki* produced smaller litters, but larger offspring, likely trading off offspring quantity for enhanced mother survival in the face of stress. This is similar to the response of guppies (*Poecilia reticulata*) to food stress [42]. Alternatively, the *G. hobrooki* mothers simply produced larger offspring that were more likely to survive when predators are around, i.e. optimizing offspring characteristics rather than degraded reproduction due to stress. The lack of response of *H. formosa* to predator treatment suggests resistance or insensitivity to stress. Reznick et al. [29] suggested that matrotrophic *H. formosa* were less able to modify offspring size in response to food level variation than lecithotrophic species such as *P. reticulata*. Since lecithotrophic species such as *G. holbrooki* must invest energy into offspring nourishment before fertilization, they may be more sensitive to stress effects than matrotrophs (i.e. after eggs have been yolked, 21, 43]. The fitness consequences of plasticity in response to stress are beyond the scope of this study, but are important to fully explore the implications of PIBS.

Differences in the response to predators can also be linked to how stress in transferred and regulated in the offspring. A positive relationship between maternal and egg glucocorticoid (stress hormone) concentrations have been found in fishes [44], [45]. Though we did not measure stress hormone levels in mothers or offspring, we hypothesize that prey individuals exposed to predators produce higher levels of stress hormones, which altered metabolic costs of the developing embryos. Cortisol influences egg metabolic rate because it has a strong positive effect on metabolic rates in adult fishes [46]. Early exposure to cortisol can also influence egg size and embryo survival [44], and also influences growth and development [47] and behavior [48]. Future research should examine differences in the role of cortisol in regulating lecithotrophic and matrotrophic investment in prey offspring. Matrotropy may allow mothers to directly regulate (and reduce) stress levels in offspring. Hence, *H. formosa* may have transferred less stress to their offspring than the lecithotrophic *G. holbrookii*. Additionally, superfetation (i.e. clutch overlap), though not quantified in this study, may have also helped reduce the predatory stress effects on reproduction in the matrotrophic *H. formosa*. Thus, matrotrophy, superfetation, or both may allow mothers to better respond to environmental fluctuation in both resource (food) and stress (predation risk) levels than lecithotrophy.

There is a possibility that the difference in response between the two species is an artifact of the season when the experiments were conducted. *G. holbrooki* was tested during its prime breeding period, while *H. formosa* was tested later in the season at a time when natural populations of the species have either stopped breeding or are slowing considerably. Even though the conditions during the experiment were constant (day length and temperature), *H. formosa* individuals might still have been primed to expect a cessation of breeding. As noted above (see introduction), due to the life-history tradeoff between breeding effort and parental survival, at the end of a breeding season there is little future gain to withholding reproductive effort, even in the face of predation risk, because few females who are sexually mature at the start of winter survive to reproduce the following spring [31], [49], [32]. Future studies should not only test *H. formosa*’s response during peak breeding season, but also *G. holbrooki*’s response later in the season.

Differences in reproductive strategy may also explain why *G. holbrooki* produced fewer and larger offspring under risk, while *H. formosa* did not. Since predation risk favors reduced reproductive allocation in both species, we expect stronger tradeoffs between offspring size and number in primarily lecithotrophic species such as *G. holbrooki* simply because the majority of investment is primarily made prior to fertilization at yolk, while in primarily matrotrophic species such as *H. formosa*, investment is spread over time and effects of stress on embryos can be adjusted throughout gestation. For *G. holbrooki*, which lacks superfetation and has relatively little post-fertilization maternally supplemented nourishment, this trade-off (offspring size vs number) may also be mediated by the ovarian space, and embryo packing determined at or soon after the yolking stage [43]. Generally, predation risk declines with body size [50] and larger individuals are better in their escape abilities [51]. For example, female sticklebacks (*Gasterosteus aculeatus*) have been shown to produce larger eggs (with high cortisol level) when exposed to predation [23], and this may be similar to what we found in *G. holbrooki*, if egg size is positively correlated with offspring size. Superfetation in *H. formosa*, on the other hand, may alleviate space constraints in the ovary [52], [53], leading to the differences in their life-history response to predation risk that we observed. Identifying the constraints on matrotrophy and evaluating it’s ‘adaptiveness’ remain a topic of debate [54].

Significantly higher proportion of stillbirths in predator exposed *G. holbrookii* compared to controls is consistent with a stress response, while the lack of differences in the matrotrophic *H. formosa* suggests a better ability to regulate stress in the developing offspring. Since the dead offspring were fully developed, these represent stillbirth (embryo death occurred in an advanced stage of development) rather than miscarriage (embryo death occurred in an early stage of development). These results are novel. Though previous experiments have shown that predation risk influences parental care (leading to greater proportion of failed hatchlings) and clutch size in prey organisms [e.g. 13], to the best of our knowledge no experimental study has shown predation risk to affect the number of stillbirths in a prey organism. This indicates yet another way that predation stress may affect population size of some prey species. The above results are also important in the context of the evolution of the placenta. According to the Trexler–DeAngelis [43] model, placentas should evolve in environments with consistently high levels of resource availability. An assumption highlighted by this model is that placental species abort embryos in low food conditions. This is the adaptive hypothesis, which assumes that the placenta evolves in response to some external ecological selection pressure in the environment [55], [43]. A higher rate of stillbirths in predator-exposed *G. holbrooki* indicate that predation risk can also be an important external selective force (like food), with the potential to drive the evolution of the placenta in vertebrates.

We hypothesize that there is a relationship between mode of reproduction and response to predation risk, and future studies should test similar effects on several prey species, from each of the continuum of reproductive modes (oviparous, lecithotrohic and matrotrohic). Studies should not only test for the presence of PIBS in other predator-prey systems, but also conduct studies over a longer period to understand if PIBS is a short or long term life-history response. Ideally, these studies should also measure physiological and behavioral responses of prey [e.g.3, 4, 23] since they are vital for understanding the underlying mechanisms of these stress responses. Incorporating predation risk into life-history theory will not only provide important insights into life-history evolution [56], [57], but it will also help in understanding the implications of maternally-derived stress in an ecological context [58] and aid in elucidating the ecological impacts of changes in predator populations.
